# Lived Experiences of Urine Drug Testing Among Individuals with a Substance Use Disorder: A Punitive or Supportive Intervention?

**DOI:** 10.3390/nursrep16020038

**Published:** 2026-01-23

**Authors:** Rob van Vredendaal, Simon Venema, Sonja Kuipers, Nynke Boonstra, Symen Kornelis Spoelstra

**Affiliations:** 1 Addiction Care North Netherlands, Laan Corpus den Hoorn 102, 9729 JR Groningen, The Netherlands; rob.van.vredendaal@ggzfriesland.nl (R.v.V.); s.venema@vnn.nl (S.V.); k.spoelstra@vnn.nl (S.K.S.); 2 Research Group Addiction Sciences & Forensic Care, Hanze University of Applied Sciences, Zernikeplein 7, 9747 AS Groningen, The Netherlands; 3 Research Group Healthcare & Innovation in Psychiatry, Department of Healthcare, NHL Stenden University of Applied Sciences, Rengerslaan 8-10, 8900 CG Leeuwarden, The Netherlands; nynke.boonstra@nhlstenden.com; 4 University of Applied Science GGZ-VS, Catharijnesingel 56-1, 3511 GE Utrecht, The Netherlands; 5 UMC Utrecht Brain Center, University Medical Center Utrecht, Heidelberglaan 100, 3584 CX Utrecht, The Netherlands; 6 KieN Early Intervention Service, Oosterkade 72, 8911 KJ Leeuwarden, The Netherlands

**Keywords:** urine drug testing, substance use disorder, nursing, therapeutic relationship, empowerment, accountability, autonomy, supervised living facility

## Abstract

**Background/Objectives**: Urine drug testing (UDT) is a core component of nursing interventions within the treatment of substance use disorder (SUD). Beyond the detection of psychoactive substance use and medication adherence, UDT also provides opportunities for therapeutic dialogue, patient support, and recovery monitoring. Despite its routine use, little is known about how patients experience UDT and its potential as a therapeutic nursing tool within recovery-oriented care. This study aimed to explore patients’ lived experiences with UDT to understand its role in recovery-oriented addiction treatment. **Methods**: A phenomenological study with in-depth, semi-structured interviews was conducted among 12 residents of a supervised living facility at Addiction Care North Netherlands. Data were analyzed using Colaizzi’s seven-step method. **Results**: Four main themes were constructed in relation to trust within the therapeutic relationship—empowerment, accountability, and autonomy. Patients stated that their perception of UDTs as either supportive or punitive depended strongly on the level of trust within the therapeutic relationship. When trust was present, UDTs were experienced as supportive nursing tools that fostered empowerment and positive self-image, reinforced accountability for recovery goals, and upheld autonomy in decision-making. Conversely, in the absence of trust, UDTs were often perceived as punitive, coercive measures that undermined self-confidence and diminished accountability, ultimately hindering recovery progress. Nursing practices that emphasized nonjudgmental interpretation of results, collaborative decision-making, and patient-centered support contributed to positive experiences. **Conclusions**: Patients’ experiences indicate that the therapeutic value of UDT is highly dependent on the quality of the patient–nurse relationship. Nurses play a key role in ensuring that UDT is used as a supportive intervention rather than merely a control measure. Integrating UDT into holistic, recovery-oriented care can foster engagement, empowerment, and a sense of accountability. Future research should investigate nursing-led strategies to optimize UDT implementation tailored to treatment phase and patient needs.

## 1. Introduction

Urine drug testing (UDT) plays a critical role in addiction care, particularly in the assessment and treatment of substance use disorder (SUD). Its applications are multifaceted, including the objective detection of psychoactive substance use, monitoring of adherence to prescribed (opioid) regimens, evaluating treatment effectiveness, and assessing recovery trajectories [1,2,3]. UDT is especially valuable because clinical examination, patient self-reporting, and collateral information often underestimate the extent of substance use [4]. The American Society of Addiction Medicine recommends the integration of UDT with self-reported substance use while acknowledging the limited empirical evidence supporting the effectiveness of routine testing in improving treatment outcomes [2].

UDT is widely recognized for its cost-effectiveness, feasibility, and rapid results, as well as the ability to detect a wide range of illicit and addictive prescription drugs [5]. UDT when appropriately applied, can support clinical decision-making and enhance communication. However, inappropriate or routine use without clear clinical indication may result in stigma, disproportionately affecting marginalized populations [6]. Jain (2004) argues that professionals consider UDT as the gold standard from medical, ethical, or control-oriented perspectives, as it represents the most objective method, particularly given that self-reporting is not always reliable [7,8]. The optimal frequency for UDT, whether random or scheduled, varies depending on individual risk factors, clinical objectives, and treatment stage [3].

In clinical practice, UDT can be applied in both a punitive and therapeutic manner. UDT may function as a sanction tool to enforce substance-free environments during recovery [2]. Historically, the use of UDT has been rooted in a criminal justice approach aimed at enforcing abstinence and “catching” people who engage in illicit substance use, often resulting in punitive consequences [9]. Simultaneously, UDT can act as a boundary-setting tool, reinforcing treatment guidelines and supporting a structured recovery process [10]. Moreover, UDT is also a key component of contingency management programs, where test results are linked to rewards or consequences to reinforce positive behaviors and discourage substance use [11]. Such programs aim to promote behavior modification by offering immediate and tangible feedback to patients.

As with any diagnostic intervention, the overarching objective of UDT is to inform clinical decision-making and enhance patient care [3]. The literature demonstrates its utility in identifying undisclosed substance use, though its direct influence on treatment decisions appears limited [10]. A systematic review further highlights the lack of strong evidence supporting the role of UDT in managing patients with SUD, emphasizing the need for pragmatic intervention research using objective assessment criteria to evaluate its clinical utility [12]. Additionally, no universally accepted standards currently exist for drug testing in SUD diagnosis, treatment monitoring, or recovery support, and empirical evidence regarding its impact on clinical outcomes remains scarce [2]. Importantly, UDT may be experienced as distressing or even traumatic, especially for individuals with a history of sexual abuse, involvement in the justice system, and exposure to violence [13].

Despite these concerns, UDT is often considered to be most effective when used as a supportive and motivational tool rather than a method of control or punishment [2]. However, patients’ perspectives of UDT are not well understood. Despite its widespread use, limited research has explored how individuals with SUD personally experience UDT [13]. Most studies have focused on its role in monitoring treatment adherence and progress, with few examining its potential influence on treatment outcomes [2]. Addressing this gap is critical, as gaining insight into patients’ perspectives may provide a deeper understanding of the impact of UDTs on treatment and recovery. A recent systematic review of nursing interventions in patients with SUDs highlights the scarcity of strong evidence regarding non-pharmacological strategies such as UDT and follow-up, underscoring the need for rigorous, pragmatic studies to evaluate the clinical utility and therapeutic integration of tools like UDT [14]. Within addiction care, nurses, as frontline professionals, play a central role in shaping how UDT is administered, communicated, and integrated into the therapeutic relationship.

Furthermore, linking the present study to established substance use-related conceptual models of the treatment process, such as motivational, behavioral, and cognitive–behavioral models, strengthens its rationale. UDT can be understood not only as an assessment tool but also as a potential mechanism to reinforce motivation, provide immediate feedback, and facilitate cognitive–behavioral reflection on substance use patterns, triggers, and coping strategies. By situating UDT within these theoretical frameworks, the study highlights how patients who lived through testing may interact with broad recovery processes and therapeutic interventions [15,16]. Accordingly, this study explores how patients with SUD experience UDT in a supervised residential facility in the Netherlands and which factors shape whether UDT is perceived as supportive or punitive.

## 2. Materials and Methods

### 2.1. Study Design

This study employed a phenomenological research design to explore and understand the lived experiences of participants [17]. Phenomenology was chosen because it emphasizes the subjective experiences of individuals and aims to capture the essence of their interactions with a phenomenon—in this case, UDT. This approach allows for an in-depth understanding of participants’ perspectives and the meanings they ascribe to their direct experiences [17,18].

### 2.2. Participant Selection

The study population consisted of adult patients with SUD (≥18 years). Participants were recruited in the supervised living facilities of Addiction Care North Netherlands. The inclusion criteria for this study encompassed patients who were proficient in Dutch and willing to participate in the study. Patients who were intoxicated at the time of recruitment were excluded. Following a presentation about the study by the researcher (RVV), participants were recruited through their therapists. Those who were willing to take part in the study could express their interest to their therapist, who then informed the researcher. Subsequently, participants were contacted by phone to schedule an appointment. To facilitate participation in the study, participants were allowed to choose the time and location of their interview.

### 2.3. Data Collection

Data collection took place in 2019 at two supervised living facilities of Addiction Care North Netherlands. In these supervised living facilities, individuals struggling with SUD and psychosocial issues are provided with recovery-focused care and treatment. Treatment follows a three-phase approach: the first phase focuses on recovery across various life areas, the second focuses on stabilization and further recovery, and the third phase focuses on promoting autonomy and independent living.

Data were collected through in-depth interviews [19], focusing on the participants’ experiences and the meaning they attributed to them. The interviews were conducted by a Master of Advanced Nursing student (RVV), who was in the final phase of his studies, under the supervision of the research team (SAK, NB). Prior to the in-depth interviews, participants provided explicit consent through an informed consent form, and their anonymity was ensured. During the interview, we explored participants’ experiences with UDT, followed by open-ended questions to further explore their experiences. An aide-mémoire (Table 1) was used as a guide during the in-depth interviews and included topics such as the method of testing, presentation testing in the recovery process, UDT, self-reporting, and the effects of substance use monitoring. Observations during the interviews were recorded using memos [19].

### 2.4. Data Analysis

The interviews were audiotaped and transcribed verbatim. Data were analyzed using Colaizzi’s seven-step phenomenological approach, which allowed for both description of participants lived experience and the interpretation of the core meanings and insights they attributed to these experiences [21]. During data familiarization, transcripts were carefully read and reread, and statements that contributed to the research objective were coded using open and inductive coding. While the orientation toward the data was primarily inductive, sensitizing concepts from literature were used as a guide, reflecting a hybrid approach. Interview transcripts and codes were then further analyzed, clustered, and interpreted across the dataset. ATLAS.ti 7.2.0 (https://atlasti.com) was used for the analysis. During the data analysis phase, bracketing was employed using a reflective log and the deliberate identification and suspension of personal assumptions and preconceptions to ensure an open, unbiased, and transparent analytic process. Peer debriefing (RVV and SAK) was conducted following each step in the coding process.

### 2.5. Rigor and Trustworthiness

Various steps were taken to ensure the rigor of the analytic process by giving attention to the following trustworthiness criteria of qualitative research: credibility, dependability, confirmability, transferability, and authenticity [22,23]. The results from the literature review were incorporated into the aide-mémoire as sensitizing concepts [24], which helped to maintain consistency among participants’ responses, thereby strengthening dependability and confirmability. A pilot interview was analyzed for content and interview style to ensure the researcher remained open to participants’ meanings. The researcher’s perspectives and assumptions were acknowledged and reflected upon throughout the analysis to enhance reflexivity, as phenomenological analysis recognizes that the researcher’s subjectivity is an inherent part of meaning-making.

Efforts were made to interview participants in a way that covered a wide range of participants’ experiences. To increase transparency, we provide a detailed description of the research site and study participants. Credibility and dependability were enhanced by transcribing interviews verbatim and by using peer review to code the data, reach consensus on meanings, and establish coherence and fundamental themes. Any disagreements among codes and themes were discussed and integrated into the final codebook. The verbatim-transcribed data, along with interview condition memos, provided an overview of participants’ experiences [17]. A member check was used to confirm that the analyzed data were interpreted correctly [25].

After ten interviews, no new codes were constructed, indicating data saturation. To confirm this, two additional interviews were conducted, which did not yield new codes. The results of the study are supported with participant quotes to increase authenticity [17]. Given the narrowly defined focus of the research question and interviews, the collected data contain sufficient information to answer the research question [26].

### 2.6. Ethics

The study was conducted in accordance with the Declaration of Helsinki and approved by the scientific committee of Addiction Care North Netherlands (reference number 201931; date of approval: 1 December 2019). Participants were fully informed about the study’s purpose through informed consent, were given the chance to ask questions, and provided their written consent [19]. In accordance with international standards for data protection, data was anonymized and securely stored in the Data Stations Social Sciences repository. 

## 3. Results

A total of four women and eight men were interviewed. All interviews were held at the supervised living facility. Each interview had a duration of 45–70 min (mean 50 min). Table 2 presents an overview of the participants’ background characteristics. Four participants were in the 18–30 age group, three were in the 30–40 group, one was in the 40–50 group, three were in the 50–60 group, and one was in the 60–70 group. Of the 12 participants, 3 used alcohol exclusively; 3 used both hard drugs (e.g., cocaine, heroin) and alcohol; 5 used only hard drugs; and 1 participant used a combination of soft drugs (e.g., cannabis) and hard drugs. Four participants were in phase 1, four were in phase 2, and four were in phase 3 of treatment. At the time of the interview, the average duration of their stay was 8.6 months, ranging from 3 to 16 months.

Out of a total of 36 patients, 16 initially signed up to participate in the study. Ultimately, 12 participants agreed to take part. Two individuals could not be reached and two did not meet the inclusion criteria, one due to a relapse and one due to insufficient progress in their treatment.

A total of 31 codes in relation to UDT were constructed. Based on our analysis of the codes, we identified four factors that determine whether patients experience UDT as a supportive therapeutic intervention or a form of punishment: (1) trust in the therapeutic relationship; (2) patient empowerment; (3) accountability; and (4) autonomy. We developed four themes that demonstrate the close interplay between these factors in patients’ experiences of UDT. A key feature of the analyses was that these factors were highly intertwined.

### 3.1. Theme 1: “I Trust You Enough to Know That if You Were Using, You Would Have Told Me”: Trust in the Therapeutic Relationship as a Foundation for Experiences of Urine Drug Testing

Participants described that the trust they developed with their mental health care providers was the key factor that determined whether they experienced UDT as supportive or punitive. When coupled with trust in the therapeutic relationship, UDTs were experienced as a positive factor in patients’ recovery. However, when coupled with distrust, UDTs were experienced as punitive. Among patients who experienced trust within the therapeutic relationship, UDTs were described as more than just a clinical requirement. UDTs became integrated into the therapeutic relationship and rooted in mutual respect. This trust allowed participants to break free from the cycles of secrecy and shame that are often associated with substance use and UDT, fostering an environment of honesty. The interplay between UDT and trust in the therapeutic relationship is evidenced by the following statements from participants:

*“Yeah, I think for me it’s more about trust. You come here, make agreements with people who want to support you in every way. And then with a positive UDT you throw away that trust.”*

[P1].

*“What I find really nice is that my personal counselor says: “You don’t have to do any urine tests for me, because I trust you enough to know that if you were using, you would have told me.”*

[P12].

Trust was experienced as a cornerstone of the therapeutic relationship and their experiences of UDT, as illustrated by one participant: *“I also learned in the clinic that honesty and trust are number one in staying off drugs.”* [P8]. Participants noted that a strong therapeutic relationship made it easier to seek help, even during moments of craving. Trust was experienced to foster an environment where patients could feel safe expressing their thoughts and feelings, enabling open discussions about challenges and setbacks. This environment contributed to preventing relapses and a positive UDT. Many participants highlighted that a calm and understanding approach regarding a positive UDT fostered a sense of safety and encouraged continued transparency, as illustrated by the following interview excerpt:

*“Without judgment. No angry reaction or anything like that, just a calm and composed response. No panic, you know?”*

[P2].

Participants valued knowing that even if they had a positive UDT, their providers would respond without judgement and with understanding and support, rather than judgment or rejection. This dynamic deepened the trust between participants and their providers, reinforcing the role of UDT as a collaborative tool for sustaining long-term recovery.

*“And you always expect a certain reaction from others. Because you’re used to it, especially when you’re deep into your use, you always see a certain look from people. You know, the disappointment and all that. So, you get used to people reacting that way, and you expect that reaction when you use. It feels like a natural response. But if that reaction doesn’t come, it makes you feel good.”*

[P2].

Conversely, participants described the damaging effects of distrust relating to UDT and therapeutic relationships. When they felt mistrusted, even after making progress, it undermined their confidence and created a barrier to effective communication. One participant described: *“It was pure distrust, really. They even said: ‘I just don’t trust you anymore.’ So, it’s really nice when someone says: ‘I trust you.’”* [P12].

*“If you tested positive, you’d be judged for it right away, you know? Either a time-out, a red card, or, you know, things like that.”*

[P12].

### 3.2. Theme 2: “I Will Start Believing in Myself More”: Urine Drug Tests as Contributor to Empowerment

The second theme highlighted that receiving negative UDT results often evoked feelings of empowerment and personal achievement among participants, leading to increased self-confidence. Negative UDTs served as tangible proof of participants’ ability to regain control over their substance use. However, this was only the case when UDTs were coupled with a high degree of trust in the therapeutic relationship. 

Before treatment, many patients described experiencing diminished self-worth and feelings of unreliability stemming from their struggles with substance use, as evidenced by the following interview excerpt: *“my self-awareness and self-belief deteriorated completely*.” [P1]. Achieving negative UDT results allowed participants to demonstrate honesty about their substance use. The validation of consistent negative UDT results became a symbol of personal success, leading participants to feel empowered and motivated to maintain their progress, and reinforced patients’ confidence.

*“And when you eventually see the form, and all the urine tests are listed in a row and they’re negative, then of course that feels like a success.”*

[P12].

Participants also stressed the interplay between self-confidence, trust and UDTs throughout their treatment. As treatment progressed, many participants experienced an increase in trust in the therapeutic relationship, which often led to reduced UDT frequency and a shift toward self-reporting. One participant described, *“Well, they saw that my previous UDTs were clean, my behavior was normal, and somehow I gained their trust.”* [P5]. A reduction in UDTs combined with greater reliance on self-reports of substance use, in turn, contributed to increased self-confidence.

However, those participants whose UDT schedules did not change despite consistently negative results reported frustration and felt undervalued by constantly having to perform UDTs. For these patients, the lack of recognition from their care team undermined their accomplishments and negatively impacted their self-image, as evidenced by the following interview excerpt:

*“You know you’re doing well (…), and they don’t trust you, that’s just not nice.”*

[P5].

### 3.3. Theme 3: “It Keeps Me on Track, and It Keeps Me from Slipping up”: Urine Drug Testing as a Tool for Accountability in Recovery

Participants perceived UDTs as a valuable tool for fostering accountability in their recovery journey. Participants described how UDTs contributed to taking responsibility for their own actions and holding themselves accountable to mental health care providers, significant others, and to themselves. UDTs enhanced participants’ recognition of the consequences of their choices, both positive and negative, and ensured that their commitments were upheld. Participants highlighted how UDTs helped them implement strategies to maintain abstinence. This sense of accountability motivated participants to proactively use interventions, such as UDT or self-reporting, as safeguards against relapses. This was evidenced by participants in the following interview excerpts:

*“It’s about myself. This [UDT] is for my own benefit. I also believe it’s my responsibility.”*

[P10].

*“All the urine tests I’ve done, I’ve made sure they are saved for me. And they’ve all been clean. I take them with me, I can show them, like, “Look, I’ve kept this up for a year, and I’m still clean. Here’s all my proof.”*

[P9].

For many participants, UDTs created a structured environment that reinforced accountability to themselves, as illustrated by the following interview excerpt: *“I just want to do my best. That way, I’m doing it right for myself too, you know? I think I need some kind of validation or something”* [P5]. Others described how UDTs served to prove their commitment to recovery to their community or fellow residents, as illustrated by the following participants:

*“I feel like I have a certain responsibility toward my fellow residents. They shouldn’t have to suffer if I’ve used. You know what I mean? It’s a kind of respect.”*

[P1]

*“I just want to prove to myself, and to other people—family, friends, and everyone else.* […] *It’s like, if I do something now, I’m only messing things up for myself.”*

[P3]

Some participants also used negative UDT results as tangible proof of their commitment, helping to restore trust in relationships and demonstrate progress to external parties, such as child welfare agencies or courts, as illustrated by the following statement: *“In relation to my son and child welfare. When I deal with that in court soon, I want to be able to show a negative UDT”* [P6].

### 3.4. Theme 4: “We Made Agreements About Urine Drug Testing Together”: Autonomy and Shared Decision-Making About Urine Drug Testing Enhance the Recovery Experience

The last theme centered on the role of autonomy in participants’ decisions to use either UDTs or self-reporting during their recovery process. Having control over this choice played a crucial role in reinforcing their commitment to abstinence and enriched the recovery experience. When participants experienced greater autonomy in use of UDTs, they described greater ownership of their recovery, viewing monitoring tools like UDTs as supportive mechanisms rather than instruments of external control. A key element was the practice of shared decision-making. Participants valued being actively involved in consideration about the use and frequency of UDT or self-reporting. This collaborative process allowed monitoring strategies to be tailored to patients’ individual needs and preferences, increasing their comfort and commitment to the process, as illustrated by the following interview excerpts:

*“You can decide for yourself how often you want it. But sometimes they’ll say, ‘hey, today you’re doing a urine test’, and you just have to do it.”*

[P2].

*“When I first came here, they discuss with you how you feel about urine tests, and you make agreements about that. Some people say they want a test every few days, while with others, they agree that it will happen randomly, without notice. That’s what I chose.”*

[P3].

Participants appreciated being heard and having their opinions respected. Flexibility in determining the frequency and method of testing, such as opting for random tests instead of regular ones, further reinforced their sense of control:

*“Well, it depends. I decided with my personal counselor not to do tests every week or every other week, but instead randomly, because I know myself.”*

[P8].

*“It’s nice when you’re listened to, and they don’t just say, ‘we’re going to do it this way or that way’.”*

[P5].

Autonomy also extended to self-reporting of substance use. Participants felt they had the freedom to choose how and when to disclose cravings or relapses. One participant stated the following:

*“Because then you can’t avoid it. If they just asked me, I would’ve admitted it right away, of course. But in this case, that morning, I went straight to the office like, ‘I need to tell you something—I got a bit drunk last night [laughs].’ They said, ‘Oh, okay, let’s take a walk’, and then I told the whole story. And then it’s fine because you’re the one coming forward with it.”*

[P8].

Participants recognized that autonomy in decision-making about UDT and self-reporting reinforced a sense of trust between them and their healthcare providers. Trust and autonomy appeared to mutually reinforce each other; feeling trusted encouraged honesty and commitment to recovery, which in turn strengthened the therapeutic relationship:

*“They mentioned that they don’t feel the need to do UDTs all the time because they trust that I would tell them if I used. That gives me confidence.”*

[P5].

### 3.5. Conceptual Model of Patients’ Experiences of Urine Drug Testing in Addiction Care

**Figure 1:**
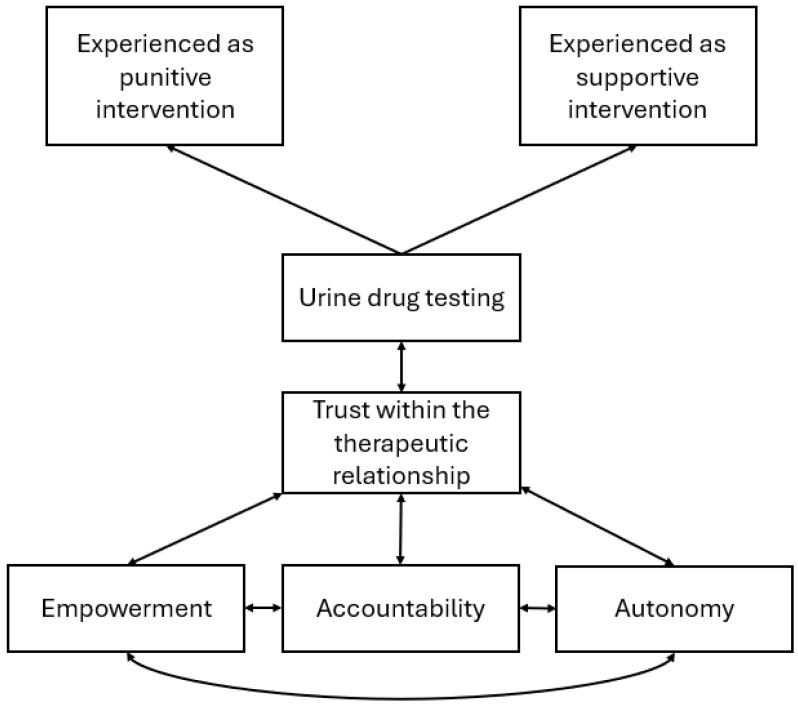
Conceptual model of patients’ experiences of urine drug testing in addiction care.

Based on our analysis, we developed a conceptual model of patients’ experiences of UDTs in addiction treatment (Figure 1). The model describes the close interplay between patients’ experiences of UDTs as a punitive or supportive intervention on the one hand, and trust in the therapeutic relationship, empowerment, accountability, and autonomy on the other. Our findings show that whether UDTs are experienced as a punitive or supportive intervention is largely dependent on trust within the therapeutic relationship. When coupled with trust, UDTs are experienced as a supportive intervention, as they promote empowerment, support accountability, and reinforce autonomy in decision-making about UDTs. However, when coupled with low trust, UDTs can be experienced as punitive. UDTs are then characterized as coercive interventions that offer little autonomy in decision-making, potentially negatively affecting participants’ self-confidence and accountability to recovery goals.

## 4. Discussion

To the best of our knowledge, this is the first study to explore how individuals with substance use disorder experience UDT within clinical addiction care from a phenomenological and recovery-orientated perspective. While previous research has primarily examined UDT in terms of clinical utility, detection, or related treatment outcomes, this study highlights patients’ lived experiences and the relational context in which UDT is embedded.

Our phenomenological analysis showed that how patients perceived UDT, either as a punitive control mechanism or as a supportive therapeutic intervention, was primarily shaped by the level of trust within the therapeutic relationship. Trust, defined as the patient’s belief that the nursing team would take the necessary actions to meet their needs and expectations, influencing disclosure, acceptance of treatment, and overall satisfaction with care, was a central mechanism through which UDT experiences were constructed [27]. This finding adds a critical relational dimension to population-based and clinical effectiveness studies, which demonstrate associations between UDT strategies and outcomes such as treatment adherence among patients with SUDs, but does not capture how patients experience these practices in everyday clinical care.

The findings of our study align with prior research showing that a strong therapeutic alliance enhances engagement and positive outcomes in SUD treatment [28,29] and highlights concerns about the detrimental impact of punitive testing approaches which can undermine trust and engagement [3,13]. In contrast to studies evaluating the technical utility of UDT [30] or concordance between self-report and UDT results [31], our findings demonstrate that the perceived legitimacy and therapeutic value of UDT depends on relational processes rather than diagnostic accuracy alone. From this perspective, UDT functions less as a neutral diagnostic instrument and more as a relational intervention embedded within recovery-orientated care.

When UDT was applied in a punitive or coercive manner, participants described experiences of surveillance, power imbalance, and reduced openness, thereby increasing the likelihood of confrontational rather than collaborative interactions [13]. These findings are consistent with concerns raised in recent debates about the elimination or reduction of routine UDT in office-based addiction treatment. Thiesen et al. (2026) reported that both patients and providers perceived routine UDT as potentially undermining trust and therapeutic engagement when framed as mandatory or disciplinary [32].

Conversely, when nurses responded to positive results with empathy, genuine interest, and a nonjudgmental attitude, this fostered interpersonal trust and psychological safety within the therapeutic relationship, allowing UDT to be experienced as a supportive and therapeutic rather than a punitive and coercive tool. These findings are in line with prior research suggesting that punitive responses may create confrontational dynamics and limit opportunities for meaningful therapeutic dialogue [3]. Under these conditions, UDT aligned recovery-oriented principles, which emphasize relational continuity, empowerment, and collaboration as core components or recovery-orientated interventions [33,34]. Participants emphasized the importance of engaging in open discussions to explore possible explanations for positive results rather than being subject to automatic sanctions.

Importantly, participants reported that supportive interpretation of UDT results enhanced their confidence in managing substance use and pursuing recovery goals. This is in line with recovery-orientated models that conceptualize monitoring practices as opportunities for reflection and skill development rather than compliance enforcement. Such empowerment may indirectly contribute to relapse prevention by strengthening individuals’ ability to anticipate and effectively manage high-risk situations [35]. Our findings are also consistent with those of Saitman (2025), who emphasized that drug testing functions best as a supportive tool within clinical care, with the interpretation of test results used to strengthen therapeutic relationships and dialogue rather than to replace them [36]

Validation of abstinence through UDT, combined with encouragement to self-report on substance use transparently, contributed to improved self-image that mitigated feelings of shame and stigma while reinforcing personal commitment to recovery. Rather than perceiving UDT as punitive or intrusive, participants experienced it as an opportunity for constructive self-reflection and responsibility-taking, thereby enhancing accountability and supporting sustained recovery. This aligns with Laudet’s (2008) assertion that recovery is strongly shaped by one’s immediate social environment; when therapeutic relationships are supportive and collaborative, tools such as UDT can function as facilitators of growth rather than mechanisms of judgment [37].

Participants emphasized the importance of actively participating in decisions regarding the implementation of UDT, which enhanced their sense of autonomy and control over their recovery process. Shared decision-making, defined as the active involvement of patients in determining aspects such as frequency, method, or its role in treatment planning, was identified as a key factor in transforming UDT from a mandatory procedure to a collaborative and participatory process. This finding corresponds with Kidd and McKinstry (2015), who argue that involving patients in treatment decisions is essential to recovery-oriented care [38]. From a nursing perspective, this emphasizes the importance of nurses facilitating shared decision-making, discussing, and negotiating monitoring plans, explaining the purpose and implications of UDT, and supporting patients to express preferences and concerns. Nonetheless, Serrano-Perez (2023) cautions that perceived control exceeding patients desired level, even within shared decision-making, may sometimes lead to an excess of responsibility, potentially adversely affecting substance use outcomes [39].

### 4.1. Limitations

Our study has several limitations. The relatively small dataset and limited sample size represent clear constraints; however, our emphasis lies in achieving an in-depth understanding and data saturation rather than statistical generalizability. The use of convenience sampling may have introduced selection bias, as only participants who were readily accessible or willing to participate were included. This potentially limits the representativeness of perspectives and experiences. In addition, the exclusion of non-Dutch-speaking participants reduced the cultural diversity of the sample, which may have affected the transferability of the findings. Moreover, the study focused on a single intervention without explicitly situating the results within broader treatment frameworks or policy contexts. A further limitation was that data collection took place within two supervised living facilities in a single region. Such settings are characterized by specific rules and expectations related to supervision and monitoring, which may differ from those in outpatient or community-based programs. This contextual factor may have influenced participants’ responses and limits the transferability of the findings to care environments. Finally, data collection relied solely on interviews, without the inclusion of complementary qualitative methods such as observations or focus groups, which could have provided additional perspectives and enhanced methodological triangulation.

### 4.2. Suggestions for Future Research

Future studies should aim to replicate this research using larger and more diverse samples. Building on the conceptual model developed, future studies could explore how the interplay of trust in the therapeutic relationship, empowerment, accountability, and autonomy influence patients’ experiences with UDTs across different clinical contexts. Further research could also explore variations in UDT experiences based on factors such as SUD type, addiction severity, treatment history, comorbidity, and socio-demographic variables. Exploring how cultural and contextual elements shaping patients’ experiences of UDT may provide deeper insight into potential strategies for enhancing therapeutic approaches. Additionally, given the importance of collaborative decision-making, examining how nurses frame UDTs, either as a therapeutic tool or a punitive measure, could offer valuable insights into more effective and recovery-oriented approaches.

### 4.3. Implications for Nursing Practice

Nurses working in supervised-living and addiction-care settings occupy a critical role in shaping patients’ experiences of UDT and thereby influencing its therapeutic potential. As frontline professionals, they are often the primary facilitators of trust, engagement, and continuity of care. Drawing on evidence from addiction nursing and therapeutic alliance research, several key nursing implications can be identified:

- Nursing training and therapeutic relationship: The therapeutic impact of UDT is strongly influenced by the quality of the patient–provider relationship [40]. Nurses should receive training focused on building trust, engaging patients in shared decision-making regarding UDT, and communicating results in an empathetical and nonjudgmental manner. Integrating UDT within recovery-oriented care frameworks supported by ongoing supervision is essential to ensure that UDT functions as a therapeutic rather than punitive practice.
- Facilitating shared decision-making around UDT: Involving patients in discussions about the purpose, frequency, and modality of UDT promotes autonomy and aligns with recovery-oriented and person-centered models of care. Nurses are uniquely positioned to negotiate these aspects collaboratively, ensuring that patients’ voices shape the process.
- Interpreting and communicating UDT results in a nonjudgmental and supportive manner: The literature emphasizes that UDT outcomes should not be used solely for sanctioning but rather as opportunities for open dialogue and reflection. By presenting results in a supportive, nonjudgmental way, nurses can transform UDT into a therapeutic encounter that informs ongoing care planning.
- Integrating UDT into recovery-oriented and empowerment-focused nursing models: When framed appropriately, negative UDTs can reinforce progress, accountability, and self-efficacy, while positive results can prompt a review of coping strategies and adjust interventions without punitive consequences. In this sense, UDT becomes part of a holistic nursing approach aimed at engaging empowerment and long-term recovery.
- At the organization level, facilities should adopt clear, flexible, and recovery-oriented UDT policies, support nurses in fostering trust and collaboration, and integrate UDT into interdisciplinary care to enhance patient engagement and ensure its therapeutic use.

## 5. Conclusions

UDT can play an important role in enhancing empowerment, accountability, and autonomy among patients with SUD. However, its effectiveness, especially from a nursing and therapeutic-care perspective, is largely dependent on the level of trust within the therapeutic relationship and the way nurses and other care professionals engage with the monitoring process. Our study underscores the critical importance of building and maintaining trust within the therapeutic relationship to ensure that UDT can function as a therapeutic tool for supporting recovery from substance use disorders.

## Figures and Tables

**Table 1:** Aide-mémoire.

Method of testing	Refers to how urine testing is conducted, including the practical aspects such as frequency, setting, privacy, communication, and manner of collection. It also includes participants’ perceptions of fairness, respect, and transparency in the testing procedure [3].
Presentation testing in the Recovery process	Describes how and when UDT is introduced during the recovery process, and whether it is framed as a supportive, motivational, or control-oriented measure within treatment [2].
UDT	The use of urine testing as an objective tool to detect psychoactive substances, monitor treatment adherence, and evaluate recovery trajectories among individuals with SUD [2].
Self-reporting	Refers to patients’ self-disclosure about substance use, typically combined with UDT to increase the validity of information on drug consumption and treatment adherence [20].
Effects of substance use monitoring	Encompasses the perceived impact of monitoring (including UDT) on the therapeutic relationship, motivation, sense of control, and recovery. Includes both positive (supportive, structured) and negative (stigmatizing, punitive) experiences [13].

**Table 2:** Characteristics of the study population.

	Gender M/F	Age Category	Substance	Phase of Treatment	Duration of Stay
1	m	50–60	Alcohol	2	7 months
2	m	30–40	Hard drugs	1	3 months
3	f	18–30	Alcohol	1	5 months
4	f	18–30	Hard drugs	2	6 months
5	m	30–40	Hard drugs and alcohol	3	14 months
6	f	30–40	Hard drugs	1	4 months
7	m	60–70	Alcohol	3	12 months
8	f	18–30	Hard drugs	1	4 months
9	m	40–50	Hard drugs and alcohol	2	10 months
10	m	50–60	Hard drugs	2	9 months
11	m	50–60	Hard drugs and alcohol	3	16 months
12	m	18–30	Soft and hard drugs	3	13 months

## Data Availability

The data presented in this study are available on request from the corresponding author due to privacy and ethical restrictions.

## Public Involvement Statement

No public involvement in any aspect of this research.

## Guidelines and Standards Statement

This manuscript was drafted against the consolidated criteria for reporting qualitative research. The core can be accessed via https://www.equator-network.org/reporting-guidelines/coreq/ (accessed on 25 March 2025).
